# Cracking the Case of Achalasia-induced Syncopal Episode

**DOI:** 10.7759/cureus.7379

**Published:** 2020-03-23

**Authors:** Paul S Bhamrah, Mehdi Faraji, Subhash Garikipati, Kenneth Ulicny, Ashley B Stutes

**Affiliations:** 1 Internal Medicine, Louisiana State University Health Sciences Center Shreveport, Shreveport, USA; 2 Radiology, Louisiana State University Health Sciences Center Shreveport, Shreveport, USA; 3 Anesthesiology, Louisiana State University Health Sciences Center Shreveport, Shreveport, USA; 4 Otolaryngology, Louisiana State University Health Sciences Center Shreveport, Shreveport, USA

**Keywords:** achalasia, syncope, asymptomatic, parasympathetic

## Abstract

Achalasia is an uncommon disease that occurs due to inability of lower esophageal sphincter from relaxing, leading to dysphagia to liquids and solids. Clues to this diagnosis include: failed treatment with proton pump inhibitors, and changes on imaging studies including chest X-ray and barium esophagogram. Ultimately it is a diagnosis made on esophageal manometry. Swallow-induced syncope has been known in patients with achalasia for almost three centuries. Here we present the case of a patient with achalasia and a non-swallowing syncopal episode. To our knowledge and extensive search, there has been no report of a similar case.

## Introduction

Achalasia is a rare condition in which the lower esophageal sphincter fails to relax, caused by inhibitory neuron degeneration. This is believed to be due to inflammation and degeneration of inhibitory neurons within the wall of the esophagus, leading to increased basal sphincter pressure and an inability of the lower esophageal sphincter to relax. The condition can be primary, where the cause is generally unknown, or secondary (pseudoachalasia), which is typically caused by some mechanical obstruction, such as a malignancy. Either type of achalasia may commonly present with progressive dysphagia to both solid foods and liquids. In even more severe cases, regurgitation, chest pain, and weight loss may also be present. This constellation of symptoms should bring to mind other possible differential diagnoses including gastroesophageal reflux disease, esophageal or peptic strictures, and cardiovascular abnormalities among others. However, the problem often lies in one of these conditions being incorrectly accepted as the diagnosis, when in fact achalasia is to blame [1].

One should suspect achalasia when a patient is having dysphagia to liquids and solids, and other treatments including proton pump inhibitors have not worked. On plain radiography, one might find nonspecific signs such as right mediastinal convex opacity, air fluid levels in the esophagus, and a small or nonexistent stomach bubble; classically, a bird beak appearance of the lower esophagus on a barium esophagogram [1]. Esophagogastroduodenoscopy would show retained food in the esophagus and resistance to the endoscopy head at the esophago-gastric transition point requiring gentle pressure to proceed further. Ultimately, esophageal manometry is the gold standard for diagnosis of achalasia, showing incomplete relaxation and aperistalsis in the lower esophagus. Treatment choice may vary from case to case, but common options include botulinum toxin injection, pneumatic dilation, or myotomy. If achalasia remains untreated for an extended period of time, it can lead to progressive dilatation of the esophagus [1].

Syncope can occur in achalasia secondary to AV nodal heart block after swallowing, which of course should involve a cardiac workup [1]. This is believed to be due to afferent impulses from the esophageal plexus which travel via the vagus nerve to the brain stem and then trigger efferent fibers to signal back through the vagus nerve to the heart. This type of syncope has been reported since 1793 by Spens, but literature review has not shown syncope to occur in achalasia without swallowing [2]. We present the case of a patient with achalasia and a non-swallowing syncopal episode. To our knowledge, there has been no report of a similar case in which a patient with achalasia that was asymptomatic had a syncopal episode not induced from swallowing.

## Case presentation

**Figure 1:**
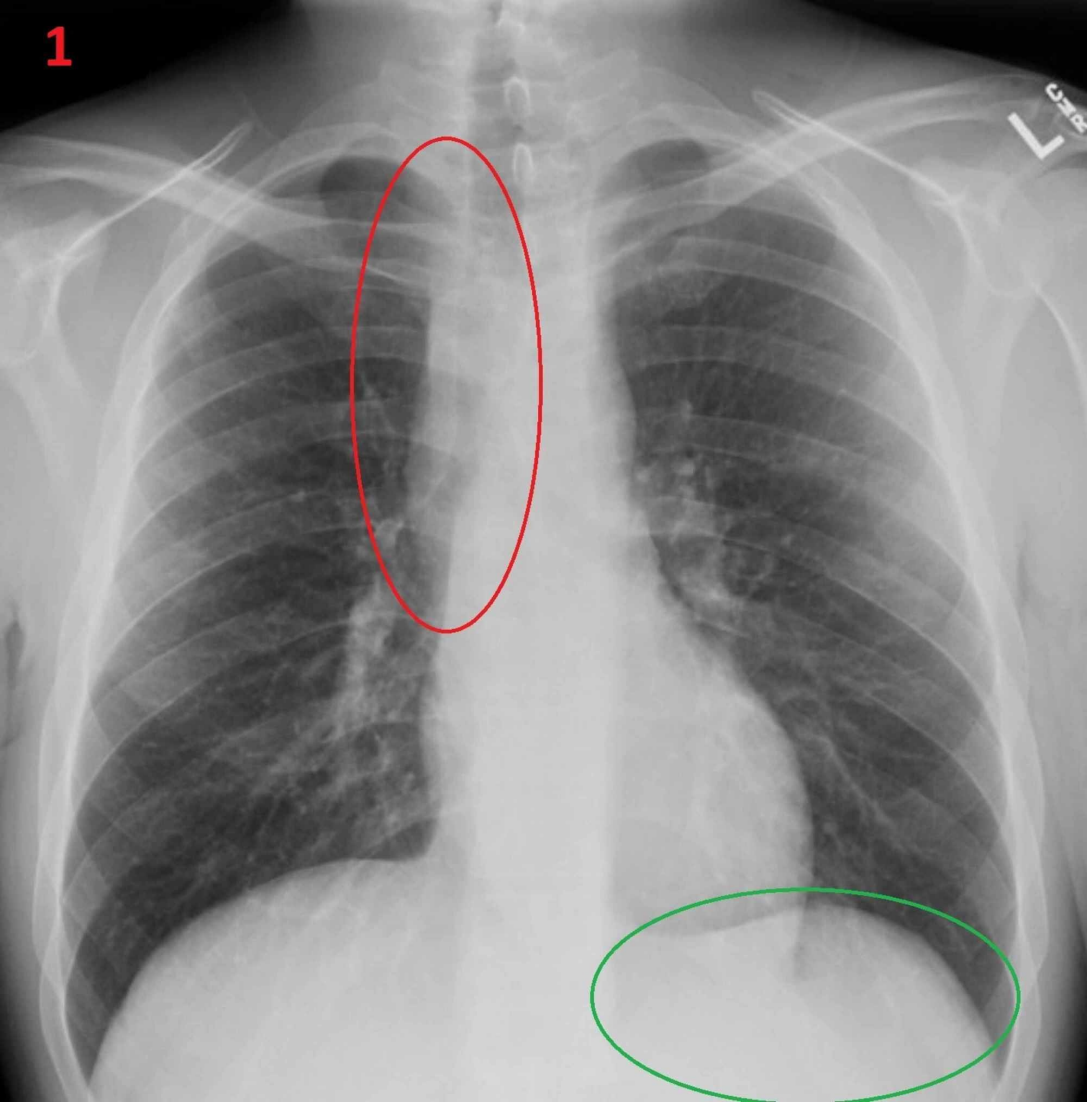
Achalasia on chest X-ray Convex opacity overlapping the right mediastinum (red oval) and no stomach air bubble on chest X-ray (green oval).

A 33-year-old man with a history of gastro-esophageal reflux, on a proton pump inhibitor, was hospitalized after having witnessed a syncopal episode, with loss of consciousness lasting 15 minutes. He states that this had occurred once before in his life. Furthermore, he mentions that in the last three years he had lost one hundred pounds and has had nausea with vomiting undigested food almost daily. The patient reported that he could not tolerate cold drinks because of chest pain on ingestion. Patient’s heart rate at presentation was 57 in sinus bradycardia and he said that his heart rate had been in the 50’s for multiple years. Chest X-ray showed convex opacity overlapping the right mediastinum and no stomach air bubble, suggestive of achalasia (Figure 1).

**Figure 2:**
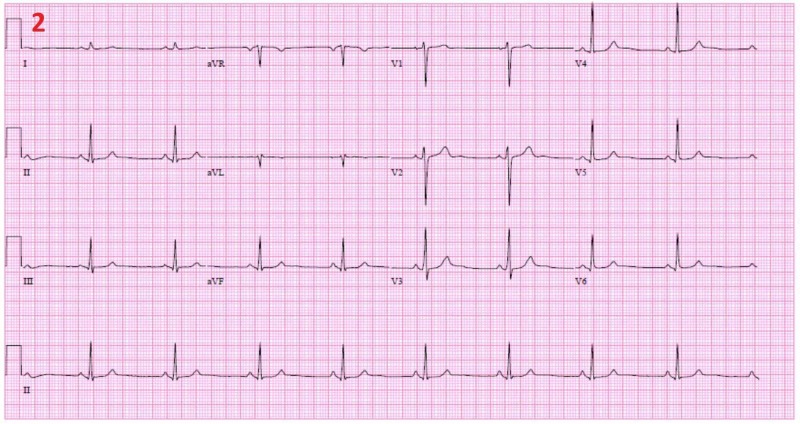
Electrocardiogram Electrocardiogram (EKG) showing bradycardia at a rate of 53 beats per minute.

Cardiac workup included electrocardiogram (EKG) showing sinus bradycardia, telemetry monitoring, and an echocardiogram which was positive for a small amount of compression of the left atrium (Figure 2).

**Figure 3:**
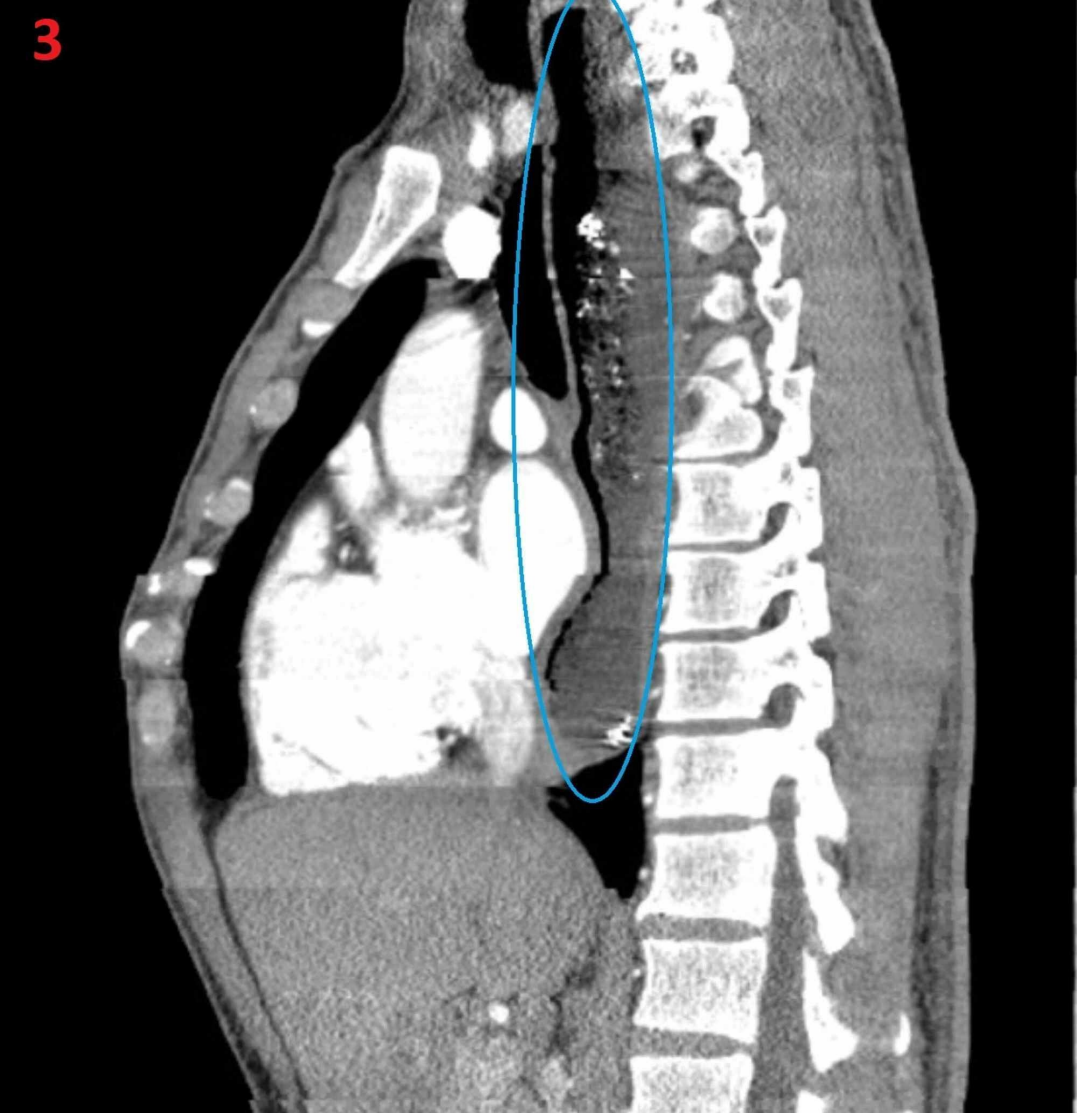
Achalasia on CT chest Mild tracheal and left atrial compression from enlarged esophagus (blue oval).

Mild tracheal and left atrial compression was seen on the CT chest (Figure 3).

**Figure 4:**
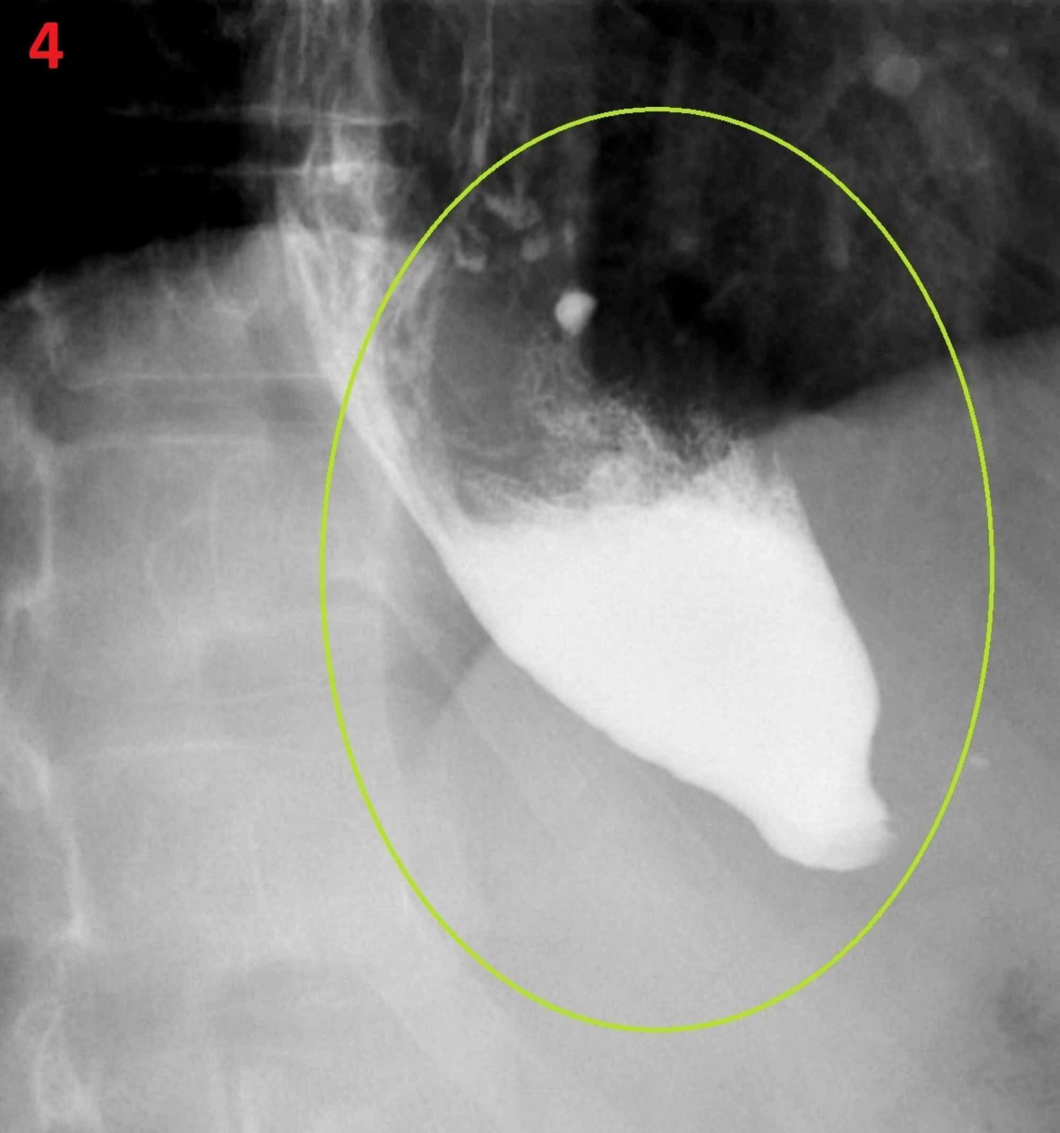
Barium esophagogram Bird’s beak accumulation of contrast in lower esophagus (yellow circle) with no trickling of contrast into the stomach.

The following day, a barium esophagogram was performed and it showed the characteristic of bird’s beak appearance without contrast trickling into the stomach (Figure 4).

On the third day, the patient had an esophagogastroduodenoscopy that revealed normal appearing esophageal mucosa, food particles retained in the esophagus, mostly liquid, which was suctioned. They also witnessed a severely dilated esophagus, and resistance to endoscopy head at the esophageal gastric transition that required gentle pressure. Manometry was needed validating achalasia. General surgery was consulted who were planning Heller myotomy on a future date. The patient was instructed to avoid cold beverages, large boluses of food, and to maintain a liquid diet.

## Discussion

Achalasia is a rare condition whose symptoms are frequently mistaken for another more common diagnosis. Therefore, many patients suffering from achalasia wait years to receive the proper diagnosis and treatment. We present such a case where this patient experienced chronic life-altering symptoms and yet went years without the correct diagnosis. Additionally, a unique feature of this case is that the patient had syncopal episode secondary to his achalasia that was not specifically induced by consumption or swallowing, rather by chronic distension of the lower esophagus and consequent bradycardia. Our patient had all the classical diagnostic findings including: proton pump inhibitors trial failure and the expected findings seen on the chest X-ray including right mediastinal convex opacity and lack of a stomach air bubble. Of course, retained food in the esophagus and resistance to endoscopy head was also seen on esophagogastroduodenoscopy at the esophageal gastric transition that required gentle pressure. The classical barium esophagogram findings were also present: bird’s beak appearance due to contrast accumulation without contrast seepage into the stomach.

This case exemplifies that syncope can occur in achalasia without swallowing, likely due to chronic pressure on the ballooned lower esophageal afferent neurons and possibly due to structural pressure on the heart itself. Syncope commonly occurs in achalasia secondary to AV nodal heart block after swallowing [1]. This syncope is a rare condition. It is thought that the abnormal reflex involves increased afferent vagus stimulation from mechanoreceptors due to esophageal distension, theoretically leading to sympathetic withdrawal and increased parasympathetic efferent innervation and resultant peripheral vasodilation, hypotension, bradycardia, and AV block. This is all thought to be due to reflex arcs in the parasympathetic system between afferent and efferent fibers [3]. Dilatational treatment of the achalasia typically results in complete resolution of the deglutition syncope [2,4,5].

## Conclusions

Achalasia is a rare case of syncope that can impair a patient’s quality of life, and can be dangerous with certain occupations, i.e., involving heavy machinery. In our literature review, we did not find a report of a similar case in which a patient with achalasia that was asymptomatic had a syncopal episode not induced from swallowing. This syncope is typically triggered by consumption of cold and/or carbonated beverages. Other triggers include viscous material and belching. The curative treatment is to treat the underlying cause, i.e., correction of achalasia. Temporizing methods can include changing dietary habit, eating smaller bolus of meals more frequently and avoiding triggers, i.e., cold beverages. The appropriate management of this condition requires a collaborative effort between diagnostic and surgical services.
